# Implication of *BRCA2 *-26G>A 5' untranslated region polymorphism in susceptibility to sporadic breast cancer and its modulation by *p53 *codon 72 Arg>Pro polymorphism

**DOI:** 10.1186/bcr1780

**Published:** 2007-10-18

**Authors:** Sailesh Gochhait, Syed Irfan Ahmad Bukhari, Narendra Bairwa, Shivani Vadhera, Katayoon Darvishi, Mohammad Raish, Pawan Gupta, Syed Akhtar Husain, Rameshwar NK Bamezai

**Affiliations:** 1 National Centre of Applied Human Genetics, School of Life Sciences, Jawaharlal Nehru University, Aruna Asafali Road, New Delhi-110067, India; 2 Human Genetics Laboratory, Department of Biosciences, Jamia Milia Islamia, Maulana Mohammad Ali Zohar Road, New Delhi-110025, India; 3 Dharamshila Cancer Hospital and Research Institute, Dharamshila Marg, Vasundhara Enclave, Delhi-110096, India

## Abstract

**Introduction:**

The absence of mutation or promoter hypermethylation in the *BRCA2 *gene in the majority of breast cancer cases has indicated alternative ways of its involvement, deregulated expression being one possibility. We show how a polymorphism in the 5' untranslated region (UTR) of *BRCA2 *can serve as one such factor. Based on the hypothesis that variants of genes involved in the same pathway can influence the risk provided for breast cancer, the status of *p53 *codon 72 polymorphism was also investigated and a possible interaction between the polymorphisms was examined.

**Methods:**

The luciferase reporter assay followed by RNA secondary structure analysis was used for the functional characterization of -26 5' UTR G>A polymorphism in *BRCA2*. The genotype and the allele frequency for the polymorphisms were determined and relative risk adjusted for age was calculated in a case-control study of 576 individuals (243 patients and 333 controls) from north India.

**Results:**

-26 G>A polymorphism in the 5' UTR of *BRCA2 *was found to be functional whereby the A allele increased the reporter gene expression by twice that of the G allele in MCF-7 (*P *= 0.003) and HeLa (*P *= 0.013) cells. RNA secondary structure analysis by two different programs predicted the A allele to alter the stability of a loop in the vicinity of the translation start site. Its direct implication in breast cancer became evident by a case-control study in which the heterozygous genotype was found to be protective in nature (*P*_heterozygote advantage model _= 0.0005, odds ratio [OR] = 0.5, 95% confidence interval [CI] = 0.4 to 0.8), which was further supported by trends observed in a genomic instability study. The *p53 *codon 72 Arg homozygous genotype was found to be over-represented in patients (*P *= 0.0005, OR = 2.3, 95% CI = 1.4 to 3.6). The interaction study indicated an increased protection under simultaneous presence of protector genotypes of both the polymorphic loci (*P *= 0.0001, OR = 0.2, 95% CI = 0.1 to 0.4).

**Conclusion:**

Our study shows that -26 5' UTR polymorphism in *BRCA2 *can modulate the fine-tuned regulation of the multifunctional gene *BRCA2 *and renders risk or protection according to the genotype status in the sporadic form of breast cancer, which is further influenced by the germline genetic backgrounds of codon 72 polymorphism of *p53*.

## Introduction

*BRCA2*, since its discovery as a breast cancer susceptibility gene [1], has been implicated in processes fundamental to all cells, including proliferation [2], development [3,4], DNA repair [5,6], transcription [7], and centrosome duplication [8]. Consistent with the tumor suppressor status of the gene, tumors that develop in carriers of heterozygous *BRCA2 *mutations are frequently associated with loss of heterozygosity at the *BRCA2 *locus [9]. Inherited mutations in the gene continue to be associated with the familial form of breast, ovarian, and other types of cancer [10,11], which represent only a small proportion of the total cases. The role of *BRCA2 *in the development of the sporadic form of breast cancer remains undefined. Although loss of heterozygosity of the *BRCA2 *locus has been detected in more than 50% of sporadic breast tumors [12], somatic mutations [13-15] or inactivation by methylation has been either absent or rare [16]. The involvement of altered expression of *BRCA2 *in the development of sporadic breast cancer is a possibility.

*BRCA2 *gene expression, owing to its functional relevance, is tightly regulated by several known and unknown factors, which in turn could be candidates responsible for the deregulated expression of *BRCA2*, thus leading to cancer. Its expression is elevated indirectly in response to the mitogenic activity of estrogen, which has been associated with progression of the cell cycle [3]. Cell cycle-dependent expression has been associated with binding of the upstream stimulatory factor protein and EIf-1 transcription factor to the *BRCA2 *promoter [17]. Wu and colleagues [18] provided evidence for direct induction of the *BRCA2 *promoter through binding of nuclear factor-kappa B. *BRCA2 *shares a complex regulatory loop with *p53 *that may be directly associated with the cellular response to DNA damage. *BRCA2 *limits the length or severity of the *p53*-mediated cell cycle-arrest phenotype by inhibiting transactivation activity of *p53 *[4,19,20], and *p53 *represses *BRCA2 *expression in response to genotoxic stress [21].

Thus, polymorphisms in the regulatory region of the *BRCA2 *gene, which can modulate the fine-tuned regulation of its expression, are logical candidates to provide risk for breast cancer. In the present study, we ascertain the functional role of a common polymorphism -26 G>A (GenBank accession number AY151039, rs1799943) in the 5' untranslated region (UTR) of the *BRCA2 *gene and test its implications in breast cancer pathogenesis. Furthermore, it has been hypothesized that breast cancer is a complex disease and that variants in multiple biologically related genes combine to affect the risk. In light of the fact that there is a functional interaction between *BRCA2 *and *p53 *in the DNA damage response pathway, the status of the widely studied *p53 *codon 72 Arg/Pro polymorphism [22-41] and its interaction with -26 *BRCA2 *polymorphism was also evaluated.

## Materials and methods

### Cloning of *BRCA2 *promoter + 5' UTR

The minimal promoter of *BRCA2 *and the 5' UTR with A or G at the -26 position (HUGO nomenclature) was amplified from already genotyped healthy control DNA, using the following primers: forward 5'-GATACTGACGGTTGGGATG-3' and reverse 5'-ATTTTTACCTACGATATTCCT-3' with long polymerase chain reaction (PCR) enzyme mix (Fermentas Canada Inc., Burlington, ON, Canada). The amplified product was cloned in TOPO cloning vector (Invitrogen Corporation, Carlsbad, CA, USA). It was then digested with Kpn and XhoI (Fermentas Canada Inc.) for cloning into pGL3 basic vector (Promega Corporation, Madison, WI, USA). Sequencing (Applied Biosystems, Foster City, CA, USA) was performed to confirm positive clones and the change at -26 5' UTR.

### Cell culture

Human breast adenocarcinoma MCF-7 and cervical carcinoma HeLa cells were propagated in Dulbecco's modified Eagle's medium (DMEM) supplemented with 10% bovine calf serum and maintained at 37°C with 5% CO_2 _(cell culture reagents were obtained from Life Technologies, Inc., now part of Invitrogen Corporation).

### Transient transfection and luciferase assays

Plasmid DNA was isolated using the plasmid maxi kit (Qiagen Inc., Valencia, CA, USA) for transient transfection. MCF-7 and HeLa cells were plated at a density of 1 × 10^5 ^cells per well in six-well plates and grown in DMEM with 10% bovine calf serum overnight, prior to transfection. All transfections were carried out using PolyFect (Qiagen Inc.) according to the manufacturer's instructions. A total of 1 μg of *BRCA2 *promoter construct and 0.1 μg of pRL-TK *Renilla *luciferase vector (Promega Corporation) with 5 μL of PolyFect were used for each transfection. The pRL-TK *Renilla *luciferase activity was used as a control for transfection efficiency. Each transfection experiment was performed in duplicate and repeated a minimum of three times. Firefly luciferase and *Renilla *luciferase assays were performed using the Dual-Luciferase Reporter Assay System (Promega Corporation). Approximately 48 hours after transfection, cells were washed twice with 1× phosphate-buffered saline and harvested with 600 μL of passive lysis buffer (Promega Corporation). Cell lysates were cleared by centrifugation, and 5 μL was added to 100 μL of firefly luciferase substrate, and light units were measured in a luminometer (TD-20/20, DLReady; Turner Designs, Inc., Sunnyvale, CA, USA, and Promega Corporation). *Renilla *luciferase activities were measured in the same tube after addition of 100 μL of Stop and Glo reagent. For adriamycin (ADR) (Sigma-Aldrich, St. Louis, MO, USA) treatment experiments, MCF-7 and HeLa cells were transfected, grown for 24 hours, and exposed to 0, 1, and 2.5 μM ADR for 1 hour in standard medium. The cells were washed with serum-free medium and incubated at 37°C in fresh culture medium for another 24 hours before harvesting for luciferase assay.

### Cases and controls

A total of 576 peripheral blood samples from north India were studied for the germline status of polymorphisms in the 5' UTR of *BRCA2 *and codon 72 of the *p53 *gene. This included 243 patients with sporadic breast cancer and 333 unrelated, healthy female controls with no history of breast cancer. In 145 of the 243 patients, tumors along with corresponding blood samples and adjacent normal tissues (only in 5 cases in which blood sample was not available) were also obtained to assess the somatic status of the mentioned amplified regions and genomic instability for the informative markers (mentioned below) established over a period of time in our laboratory in a pair study. Care was taken that both cases and controls were ethnically and geographically matched. Prior approval was given by the Jawaharlal Nehru University ethical committee and the concerned subjects for sample collection and study. The tissue samples were frozen immediately after collection and stored at -80°C until use. DNA was extracted from the peripheral blood leucocytes and from tissues according to the standard phenol-chloroform method.

### Polymorphism study

PCR was performed under conditions similar to those described earlier [42] with the following primer sequences (respective annealing temperatures are mentioned alongside): -26G>A polymorphism in *BRCA2*, forward 5'-CTC AGT CAC ATA ATA AGG AAT-3' and reverse 5'-ACA CTG TGA CGT ACT GGG TTT T-3' (55°C) and exon 4 codon 72 Arg>Pro polymorphism, forward 5'-TGC TCT TTT CAC CCA TCT AC-3' and reverse 5'-ATA CGG CCA GGC ATT GAA GT-3' (62°C). Thereafter, polymorphisms were detected by sequencing of their respective PCR products.

### Genomic instability analysis

Mutation analysis of exons 5 to 8 of the *p53 *gene and allelic imbalance study using three informative markers each in chromosome 16 (D16S3082, D16S423, and D16S413) and chromosome 17 (D17S379, D17S934, and D17S787) were carried out according to the procedure mentioned earlier [42] in 145 of 243 paired samples.

### Statistical analysis

First, overall genotype frequencies of breast cancer patients and control subjects were compared using the 3 × 2 chi-square test. Once a significant overall difference between patients and controls subjects was detected (*P *< 0.05), the individual genotypes were analyzed using an unconditional logistic regression model with correction for age using the SPSS statistical package, version 10 (SPSS Inc., Chicago, IL, USA). To compare the relative luciferase activity and the genomic instability analysis, the *t *test and the chi-square/Fisher exact test were used, respectively [43]. Statistical significance was considered at a *P *value of less than or equal to 0.05.

## Results

### Effect of 5' UTR -26 G>A polymorphism on reporter gene expression

**Figure 1:**
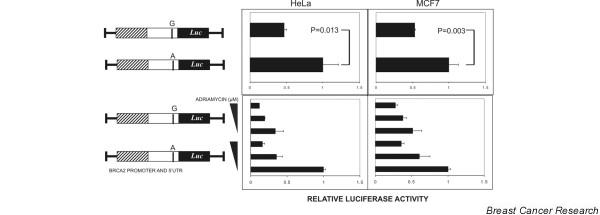
Reporter gene assay with chimeric *BRCA2 *promoter and 5' untranslated region (UTR) with A or G at the -26 position (upper panel) and the effect of increasing the dose of adriamycin (0, 1, and 2.5 μM) (lower panel).

To study the effect on expression of *BRCA2 *5' UTR -26 G>A polymorphism, HeLa and MCF-7 cells were transiently transfected with reporter gene construct (pGL3Basic) with A or G at the polymorphic site. Figure 1 (upper panel) shows how the construct containing the A allele showed significantly higher expression than the one with the G allele in HeLa (*P *= 0.013) as well as MCF-7 (*P *= 0.003) cell lines. Both HeLa and MCF-7 cells exposed to various concentrations of ADR for 1 hour after 24 hours of transfection downregulated the overall expression as expected, but the trend of A allele expressing higher than its alternative G allelic form was maintained (Figure 1, lower panel). The increased activation of *p53 *by ADR treatment was observed in our experiments as well (data not shown).

### Computational assessment of the regulation of *BRCA2 *expression by -26 G>A polymorphism

**Figure 2:**
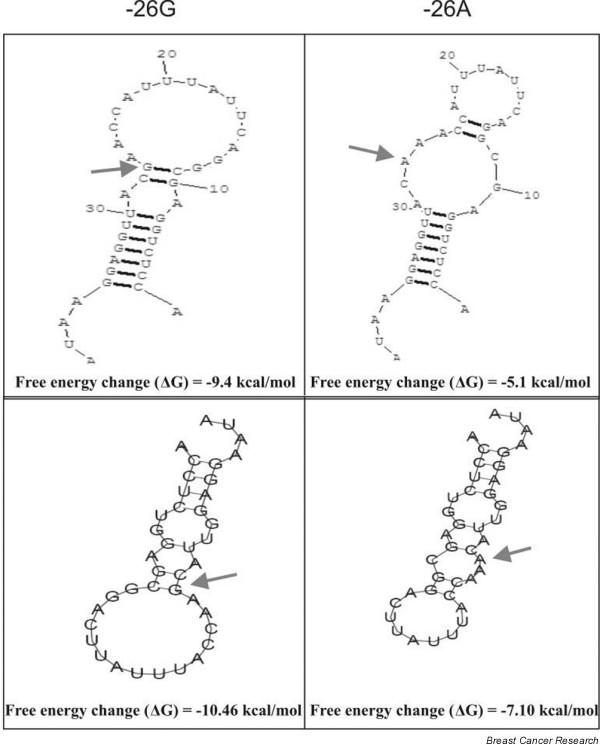
Effect of -26 G/A polymorphism on the 5' untranslated region secondary structure of RNA and stability analyzed by the RNAstructure program (version 4.3) and VRNAAFOLD (The European Molecular Biology Open Software Suite). The -26 position with G or A is indicated by arrows.

The analysis of the polymorphic region for the presence of a putative transcription factor binding site by means of the AliBaba 2.1 program [44] revealed that the G>A change creates a C/EBP alpha (TTTACCAAGC > TTTACCAAAC) binding site. The presence of an OCT1 binding site almost overlapping to this region, however, was not affected by the polymorphism. The analysis of the complete 5' UTR with G or A at the -26 position for RNA secondary structure folding by RNAstructure program (version 4.3) [45] depicted a 40-base pair (bp) loop formation in the vicinity of the translation start site and this structure changed with the change of the G>A base at the position. Furthermore, RNA secondary structure folding of the 40-bp loop with free energy change (ΔG) calculation, when compared using two different programs, showed a similar trend in the results obtained. The base substitution brought a change in the structure, and the presence of G at the -26 position significantly increased ΔG (-9.4 kcal/mole versus -5.1 kcal/mole by RNAstructure V4.3 and -10.46 kcal/mole versus -7.10 kcal/mole by VRNAAFOLD, The European Molecular Biology Open Software Suite) [46] when compared with that of the A allele at the same position (Figure 2).

### 5' UTR -26 G>A polymorphism in association with breast cancer risk

To further assess the relevance of this functional polymorphism, a preliminary case-control study was undertaken to assess the effect of -26 G>A on sporadic breast cancer susceptibility. Table 1 presents the details of the genotype and allele frequencies along with the risk calculations. The overall distribution of genotype frequencies between patients and controls was found to be significant (*P *= 0.001). Surprisingly, the heterozygous genotype provided approximately twice the protection than the common G/G homozygous state (*P*_heterozygote advantage model _= 0.0005, odds ratio [OR] = 0.5, 95% confidence interval [CI] = 0.4 to 0.8). However, there was no significant difference in the distribution of the A/A homozygous genotype in comparison with the G/G homozygous state (*P *= 0.677, OR = 1.1, 95% CI = 0.6 to 2.1) and the allele frequencies (*P *= 0.092).

**Table 1:** Genotype and allele frequencies of *BRCA2 *-26 and *p53 *codon 72 polymorphisms in sporadic breast cancer patients and normal controls

Genotypes	Patients	Controls	*P*^a^	*P*^b^	*P*^c^	OR (95% CI)^d^
	*N *= 243	*N *= 333				

*BRCA2*, exon 2, -26G>A						
G/G	144 (59.3%)	157 (47.1%)			-	1.0 (referent)
G/A	74 (30.5%)	152 (45.6%)			0.0005^e^	0.5 (0.4–0.8)
A/A	25 (10.3%)	24 (7.2%)	0.002, 0.116	0.001	0.677	1.1 (0.6–2.1)
G	74.50%	70.00%			0.092	1.2 (1.0–1.6)
*p53*, exon 4, codon 72 Arg>Pro						
Pro/Pro	48 (19.8%)	97 (29.1%)			-	1.0 (referent)
Pro/Arg	109 (44.9%)	160 (48.0%)			0.119	1.4 (0.9–2.1)
Arg/Arg	86 (35.4%)	76 (22.8%)	0.210, 0.521	0.001	0.0005^e^	2.3 (1.4–3.6)
Pro	42.20%	53.20%			0.0002^e^	0.6 (0.5–0.8)

^a^*P *value for Hardy-Weinberg equilibrium testing. ^b^*P *value for 3 × 2 chi-square test of comparison of overall genotype frequencies between breast cancer patients and controls. ^c,d^*P *values and corresponding age-adjusted ORs with 95% CIs for comparison of genotype frequencies between breast cancer patients and controls by logistic regression (not adjusted for age in allele frequency comparisons); the referent genotypes were considered on the basis of highest proportion of a homozygous genotype at any particular locus in the control group. ^e^*P *values continue to remain significant after applying Bonferroni correction of 2 (2 single-nucleotide polymorphisms tested in a case-control group) to correct for multiple comparisons. CI, confidence interval; OR, odds ratio.

### Heterozygous genotype in association with genomic instability

**Figure 3:**
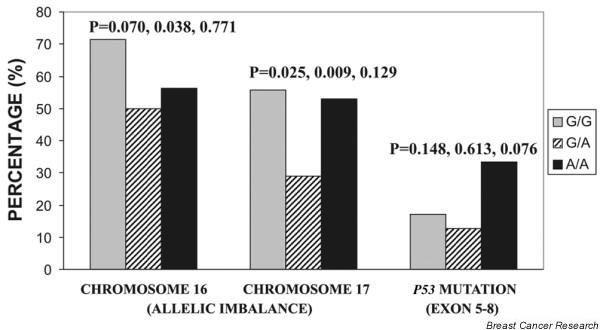
Comparison of allelic imbalance in chromosomes 16 and 17 along with mutation in *p53 *with -26 G>A polymorphism background. *P *values given are for overall distribution, G/G versus G/A, and A/A versus G/A.

To test the hypothesis that the *BRCA2 *heterozygous genotype provides protection in comparison with either of the homozygous genotypes at the -26 position in the 5' UTR, allelic imbalance for the selected informative markers in chromosomes 16 and 17 was studied along with mutation in the *p53 *gene (mutation details provided in Additional File 1) in a panel of normal and tumor tissues already characterized for the polymorphism. Interestingly, the tumors with a heterozygous background for the polymorphism showed a decreased frequency of allelic imbalance for the three informative markers each on chromosomes 16 and 17 as well as *p53 *somatic mutations when compared with either of the homozygous tumors (Figure 3). Further delineation of genotype comparisons showed that GG versus GA (*P *= 0.038, *P *= 0.009) was significant compared with AA versus GA (*P *= 0.771, *P *= 0.129) for allelic imbalance in chromosomes 16 and 17, respectively. Inversely, AA versus GA (*P *= 0.076) showed a nonsignificant trend toward association compared with GG versus GA (*P *= 0.613) when *p53 *somatic mutations were compared. Incidentally, both the 5' UTR of *BRCA2 *and exon 4 of the *p53 *gene did not show any somatic variation in tumor tissues of the studied samples.

### Status of *p53 *codon 72 Arg/Pro polymorphism and its interaction with *BRCA2 -26 *polymorphism

The overall genotype frequency distribution at the codon 72 locus between patients and controls was found to be significantly different (*P *= 0.001). The *p53 *codon 72 Arg homozygous genotype (*P *= 0.0005, OR = 2.3, 95% CI = 1.4 to 3.6) as well as the Arg allele (*P*_allele model _= 0.0002, OR = 1.7, 95% CI = 1.2 to 2.0) were significantly over-represented in patients (Table 1). Stratified genotypic data to assess the combined influence of both *p53 *and *BRCA2 *polymorphisms on breast cancer risk revealed that the G/A at the -26 position of *BRCA2 *in combination with the Pro allele at the codon 72 locus provided fivefold protection against breast cancer (*P *= 0.0001, OR = 0.2, 95% CI = 0.1 to 0.4) (Table 2).

**Table 2:** Interaction between the polymorphisms: *p53 *codon 72 and *BRCA2 *-26 in sporadic breast cancer patients and normal controls

Genotypic combination		Patients	Controls		
*p53 *codon 72	*BRCA2 *-26	*N *= 243	*N *= 333	*P*^a^	OR (95% CI)^b^
Pro/Pro	G/G	38 (15.63%)	45 (13.51%)	-	1 (referent)
Pro/Pro	G/A	7 (2.88%)	46 (13.81%)	0.0002^c^	0.2 (0.1–0.4)
Pro/Pro	A/A	3 (1.23%)	6 (1.80%)	0.476	0.6 (0.1–2.5)
Pro/Arg	G/G	59 (24.27%)	78 (23.42%)	0.686	0.9 (0.5–1.5)
Pro/Arg	G/A	38 (15.64%)	68 (20.42%)	0.166	0.7 (0.4–1.2)
Pro/Arg	A/A	12 (4.94%)	14 (4.20%)	0.983	1.0 (0.4–2.4)
Arg/Arg	G/G	47 (19.34%)	34 (10.21%)	0.120	1.6 (0.9–3.0)
Arg/Arg	G/A	29 (11.93%)	38 (11.41%)	0.754	0.9 (0.5–1.7)
Arg/Arg	A/A	10 (4.11%)	4 (1.20%)	0.087	3.0 (0.9–10.2)

^a,b^*P *values and corresponding age-adjusted ORs with 95% CIs for comparison of combination of genotype frequencies between breast cancer patients and controls by logistic regression. ^c^*P *values continue to remain significant after applying Bonferroni correction of 9 (9 combinations tested in a case-control group) to correct for multiple comparisons. CI, confidence interval; OR, odds ratio.

## Discussion

The failure of mutational inactivation of the *BRCA2 *gene to show an association with breast cancer, specifically the sporadic form, in two recent reports from India [14,15] indicated the need to further explore the involvement of *BRCA2 *in breast cancer susceptibility, with altered expression as a possibility. We propose that -26 G>A polymorphism in the 5' UTR of *BRCA2 *has a regulatory role and acts as one of the risk factors, modulating the function of *BRCA2 *in breast cancer pathogenesis, further influenced by codon 72 polymorphism in the *p53 *gene.

The functional characterization of the common G>A polymorphism in the *BRCA2 *-26 5' UTR by reporter assay revealed that the A allele led to higher expression than the G allele in both HeLa and MCF-7 cell lines. This suggests that the polymorphism might have a role in the regulation of *BRCA2 *expression. Upholding the same trend by ADR treatment further implied that the -26 polymorphism might regulate *BRCA2 *expression independent of other factors, specifically to the repression of the *BRCA2 *promoter by ADR via *p53 *activation [21]. A search for a consensus sequence for the putative transcription factor in the polymorphic site revealed the creation of a C/EBP alpha transcription binding site by the change from G>A in the -26 polymorphic position. However, the presence of an OCT1 binding site almost overlapping to this region, not affected by the polymorphism, makes the transcriptional control hypothesis an unlikely one, unless proven by the gel shift or chromatin immunoprecipitation assays. The analysis of the 5' UTR for the RNA secondary structure by two different programs showed that the presence of G at -26 stabilized the loop at the vicinity of the translation start site, which could decrease the efficiency of the translation and thereby decrease the expression of *BRCA2*. This finding of ours is supported by the classic work of Vega Laso and colleagues [47] and Kozak [48], who have argued that the extent of a hairpin negative effect on eukaryotic mRNA translation *in vivo *depends upon its stability and localization within the molecule.

The -26 G>A polymorphism in the 5' UTR of *BRCA2 *has been analyzed in some cancer types. In one study, the -26A allele was reported to show a borderline association with ovarian cancer, but similar results in breast cancer with *BRCA1 *mutation carriers were not found [49]. Another study showed a monotonic increase in the frequency of the heterozygous G/A genotype from the lowest to highest risk groups of esophageal squamous cell carcinoma from northern China [50]. Our observation supports a heterozygote-protective model, which has been well recognized in infectious diseases [51] and is consistent with the initial findings of Healey and colleagues [52] in sporadic breast cancer. An increased genomic instability in the homozygotes compared to heterozygotes substantiates the protective nature of the heterozygous genotype. The differential results of the association study as reported previously could be due to the outcome of various single-nucleotide polymorphism (SNP)–SNP and SNP–environment interactions, again modulated by population and disease type. Nevertheless, our observations of the A allele associated with higher expression and increased genomic instability via inhibition of *p53 *transactivation [4,19,20] and of the G allele with low expression impairing the DNA repair leading to genomic instability [53-55] apparently explain the risk provided by the homozygous state of the two alleles A and G to breast cancer. Our results also showed that either of the homozygous forms is associated with increased allelic imbalance or *p53 *mutations, which again is in line with the findings on consequences of aberrant expression of *BRCA2 *[4,19,20,53-55]. On the basis of these observations, it seems apparent that a germline heterozygosity status at -26 5' UTR may therefore confer a protective phenotype characterized by intermediate levels of *BRCA2 *expression and an optimal balanced DNA damage response, specifically to double-strand breaks.

To test the SNP–SNP interaction model, functional polymorphism at codon 72 of *p53 *was considered based on the emerging hypothesis that variants in many genes along related biological pathways combine to influence breast cancer risk. The two alleles of the Arg72Pro polymorphism of *p53 *differ in their biological activities. The Pro allele is associated with increased transcription of *p53 *target genes via greater binding to transcriptional machinery and thus shows higher rates of G_1 _arrest than the Arg allele [22]. In contrast, the Pro allele is less efficient in suppressing transformation (likely due to reduced apoptotic potential) than the Arg allele [22-24], which could be due to efficient binding and regulation by iASPP (inhibitory member of the ASPP family) [25]. A summary of case-control reports on the association of codon 72 polymorphism with breast cancer across the globe, including our results, revealed an inconsistency in association [26-41], again pointing toward modulations by multiple factors (Additional File 2). Our analysis of the interaction between -26 *BRCA2 *and codon 72 *p53 *polymorphism showed that the simultaneous presence of protective genotypes, Pro/Pro at the *p53 *codon 72 locus and G/A at the -26 *BRCA2 *locus, rendered fivefold protection against breast cancer and was approximately twice of what each genotype rendered alone.

## Conclusion

Our study shows that 5' UTR -26 polymorphism of *BRCA2 *might regulate its expression, albeit not at a transcriptional level, but at a post-transcriptional level affecting the translational efficiency. It is interesting to presume that the low-penetrating SNP can modulate the fine-tuned regulation of the multifunctional gene *BRCA2*, leading to the differential form of genomic instability and rendering risk to the sporadic form of breast cancer. This also provides an alternative to the involvement of *BRCA2 *in the sporadic form in which inactivation by somatic mutagenesis and methylation is known to be rare. The analysis also puts into perspective the functional implication of the dimorphic germline status of *p53 *in the background of 5' UTR of *BRCA2 *in sporadic breast cancer development and calls for more of such studies in humans to understand the epistatic effect of genetic backgrounds in the promotion and progression of the cancer.

## Abbreviations

ADR = adriamycin; bp = base pair; CI = confidence interval; DMEM = Dulbecco's modified Eagle's medium; OR = odds ratio; PCR = polymerase chain reaction; SNP = single-nucleotide polymorphism; UTR = untranslated region.

## Competing interests

The authors declare that they have no competing interests.

## Authors' contributions

SG participated in the design of the experiments, experimental data acquisition, statistical analysis, and the writing of the draft. SB, SV, KD, and MR participated in the development of the study method and coordinated the collection of data. NB participated in the design of the experiments and the interpretation of data. PG, as clinician and surgeon, participated in the acquisition and interpretation of data. SH participated in the interpretation of data and gave critical suggestions. RB conceived of the study, participated in the design of the experiments and the interpretation of data, revised the draft critically for intellectual content, and gave final approval of the version to be published. All authors have read and approved the final manuscript.

## Supplementary Material

Additional file 1 Details of the somatic mutations found in the exons 5-8 (DNA binding domain) of the p53 gene in sporadic breast cancer patients Click here for file

Additional file 2 Summary of p53 codon 72 association studies in breast cancer across the globe Click here for file

## References

[B1] Wooster R, Bignell G, Lancaster J, Swift S, Seal S, Mangion J, Collins N, Gregory S, Gumbs C, Micklem G (1995). Identification of the breast cancer susceptibility gene BRCA2. Nature.

[B2] Vaughn JP, Cirisano FD, Huper G, Berchuck A, Futreal PA, Marks JR, Iglehart JD (1996). Cell cycle control of BRCA2. Cancer Res.

[B3] Rajan JV, Marquis ST, Gardner HP, Chodosh LA (1997). Developmental expression of Brca2 colocalizes with Brca1 and is associated with proliferation and differentiation in multiple tissues. Dev Biol.

[B4] Sharan SK, Morimatsu M, Albrecht U, Lim DS, Regel E, Dinh C, Sands A, Eichele G, Hasty P, Bradley A (1997). Embryonic lethality and radiation hypersensitivity mediated by Rad51 in mice lacking Brca2. Nature.

[B5] Gudmundsdottir K, Ashworth A (2006). The roles of BRCA1 and BRCA2 and associated proteins in the maintenance of genomic stability. Oncogene.

[B6] Nagaraju G, Scully R (2007). Minding the gap: the underground functions of BRCA1 and BRCA2 at stalled replication forks. DNA Repair (Amst).

[B7] Milner J, Ponder B, Hughes-Davies L, Seltmann M, Kouzarides T (1997). Transcriptional activation functions in BRCA2. Nature.

[B8] Nakanishi A, Han X, Saito H, Taguchi K, Ohta Y, Imajoh-Ohmi S, Miki Y (2007). Interference with BRCA2, which localizes to the centrosome during S and early M phase, leads to abnormal nuclear division. Biochem Biophys Res Commun.

[B9] Venkitaraman AR (2002). Cancer susceptibility and the functions of BRCA1 and BRCA2. Cell.

[B10] Levy-Lahad E, Friedman E (2007). Cancer risks among BRCA1 and BRCA2 mutation carriers. Br J Cancer.

[B11] Couch FJ, Johnson MR, Rabe KG, Brune K, de Andrade M, Goggins M, Rothenmund H, Gallinger S, Klein A, Petersen GM (2007). The prevalence of BRCA2 mutations in familial pancreatic cancer. Cancer Epidemiol Biomarkers Prev.

[B12] Bieche I, Nogues C, Rivoilan S, Khodja A, Latil A, Lidereau R (1997). Prognostic value of loss of heterozygosity at BRCA2 in human breast carcinoma. Br J Cancer.

[B13] Lancaster JM, Wooster R, Mangion J, Phelan CM, Cochran C, Gumbs C, Seal S, Barfoot R, Collins N, Bignell G (1996). BRCA2 mutations in primary breast and ovarian cancers. Nat Genet.

[B14] Valarmathi MT, Sawhney M, Deo SS, Shukla NK, Das SN (2004). Novel germline mutations in the BRCA1 and BRCA2 genes in Indian breast and breast-ovarian cancer families. Hum Mutat.

[B15] Saxena S, Chakraborty A, Kaushal M, Kotwal S, Bhatanager D, Mohil RS, Chintamani C, Aggarwal AK, Sharma VK, Sharma PC (2006). Contribution of germline BRCA1 and BRCA2 sequence alterations to breast cancer in Northern India. BMC Med Genet.

[B16] Collins N, Wooster R, Stratton MR (1997). Absence of methylation of CpG dinucleotides within the promoter of the breast cancer susceptibility gene BRCA2 in normal tissues and in breast and ovarian cancers. Br J Cancer.

[B17] Davis PL, Miron A, Andersen LM, Iglehart JD, Marks JR (1999). Isolation and initial characterization of the BRCA2 promoter. Oncogene.

[B18] Wu K, Jiang SW, Thangaraju M, Wu G, Couch FJ (2000). Induction of the BRCA2 promoter by nuclear factor-kappa B. J Biol Chem.

[B19] Marmorstein LY, Ouchi T, Aaronson SA (1998). The BRCA2 gene product functionally interacts with p53 and RAD51. Proc Natl Acad Sci USA.

[B20] Patel KJ, Yu VP, Lee H, Corcoran A, Thistlethwaite FC, Evans MJ, Colledge WH, Friedman LS, Ponder BA, Venkitaraman AR (1998). Involvement of Brca2 in DNA repair. Mol Cell.

[B21] Wu K, Jiang SW, Couch FJ (2003). p53 mediates repression of the BRCA2 promoter and down-regulation of BRCA2 mRNA and protein levels in response to DNA damage. J Biol Chem.

[B22] Pim D, Banks L (2004). p53 polymorphic variants at codon 72 exert different effects on cell cycle progression. Int J Cancer.

[B23] Thomas M, Kalita A, Labrecque S, Pim D, Banks L, Matlashewski G (1999). Two polymorphic variants of wild-type p53 differ biochemically and biologically. Mol Cell Biol.

[B24] Dumont P, Leu JI, Della Pietra AC, George DL, Murphy M (2003). The codon 72 polymorphic variants of p53 have markedly different apoptotic potential. Nat Genet.

[B25] Bergamaschi D, Samuels Y, Sullivan A, Zvelebil M, Breyssens H, Bisso A, Del Sal G, Syed N, Smith P, Gasco M (2006). iASPP preferentially binds p53 proline-rich region and modulates apoptotic function of codon 72-polymorphic p53. Nat Genet.

[B26] Sjalander A, Birgander R, Hallmans G, Cajander S, Lenner P, Athlin L, Beckman G, Beckman L (1996). p53 polymorphisms and haplotypes in breast cancer. Carcinogenesis.

[B27] Weston A, Pan CF, Ksieski HB, Wallenstein S, Berkowitz GS, Tartter PI, Bleiweiss IJ, Brower ST, Senie RT, Wolff MS (1997). p53 haplotype determination in breast cancer. Cancer Epidemiol Biomarkers Prev.

[B28] Huang XE, Hamajima N, Katsuda N, Matsuo K, Hirose K, Mizutani M, Iwata H, Miura S, Xiang J, Tokudome S (2003). Association of p53 codon Arg72Pro and p73 G4C14-to-A4T14 at exon 2 genetic polymorphisms with the risk of Japanese breast cancer. Breast Cancer.

[B29] Papadakis EN, Dokianakis DN, Spandidos DA (2000). p53 codon 72 polymorphism as a risk factor in the development of breast cancer. Mol Cell Biol Res Commun.

[B30] Buyru N, Tigli H, Dalay N (2003). P53 codon 72 polymorphism in breast cancer. Oncol Rep.

[B31] Kalemi TG, Lambropoulos AF, Gueorguiev M, Chrisafi S, Papazisis KT, Kotsis A (2005). The association of p53 mutations and p53 codon 72, Her 2 codon 655 and MTHFR C677T polymorphisms with breast cancer in Northern Greece. Cancer Lett.

[B32] Ohayon T, Gershoni-Baruch R, Papa MZ, Distelman Menachem T, Eisenberg Barzilai S, Friedman E (2005). The R72P P53 mutation is associated with familial breast cancer in Jewish women. Br J Cancer.

[B33] Damin AP, Frazzon AP, Damin DC, Roehe A, Hermes V, Zettler C, Alexandre CO (2006). Evidence for an association of TP53 codon 72 polymorphism with breast cancer risk. Cancer Detect Prev.

[B34] Katiyar S, Thelma BK, Murthy NS, Hedau S, Jain N, Gopalkrishna V, Husain SA, Das BC (2003). Polymorphism of the p53 codon 72 Arg/Pro and the risk of HPV type 16/18-associated cervical and oral cancer in India. Mol Cell Biochem.

[B35] Mabrouk I, Baccouche S, El-Abed R, Mokdad-Gargouri R, Mosbah A, Said S, Daoud J, Frikha M, Jlidi R, Gargouri A (2003). No evidence of correlation between p53 codon 72 polymorphism and risk of bladder or breast carcinoma in Tunisian patients. Ann N Y Acad Sci.

[B36] Suspitsin EN, Buslov KG, Grigoriev MY, Ishutkina JG, Ulibina JM, Gorodinskaya VM, Pozharisski KM, Berstein LM, Hanson KP, Togo AV (2003). Evidence against involvement of p53 polymorphism in breast cancer predisposition. Int J Cancer.

[B37] Noma C, Miyoshi Y, Taguchi T, Tamaki Y, Noguchi S (2004). Association of p53 genetic polymorphism (Arg72Pro) with estrogen receptor positive breast cancer risk in Japanese women. Cancer Lett.

[B38] Mahasneh AA, Abdel-Hafiz SS (2004). Polymorphism of p53 gene in Jordanian population and possible associations with breast cancer and lung adenocarcinoma. Saudi Med J.

[B39] Tommiska J, Eerola H, Heinonen M, Salonen L, Kaare M, Tallila J, Ristimaki A, von Smitten K, Aittomaki K, Heikkila P (2005). Breast cancer patients with p53 Pro72 homozygous genotype have a poorer survival. Clin Cancer Res.

[B40] Khadang B, Fattahi MJ, Talei A, Dehaghani AS, Ghaderi A (2007). Polymorphism of TP53 codon 72 showed no association with breast cancer in Iranian women. Cancer Genet Cytogenet.

[B41] Cox DG, Deer D, Guo Q, Tworoger SS, Hankinson SE, Hunter DJ, De Vivo I (2007). The p53 Arg72Pro and MDM2 -309 polymorphisms and risk of breast cancer in the nurses' health studies. Cancer Causes Control.

[B42] Mir MM, Dar NA, Gochhait S, Zargar SA, Ahangar AG, Bamezai RN (2005). p53 mutation profile of squamous cell carcinomas of the esophagus in Kashmir (India): a high-incidence area. Int J Cancer.

[B43] GraphPad Software, Inc. http://www.graphpad.com.

[B44] AliBaba 2.1. http://darwin.nmsu.edu/~molb470/fall2003/Projects/solorz/aliBaba_2_1.htm.

[B45] Michael Zuker's home page. http://frontend.bioinfo.rpi.edu/zukerm/home.html.

[B46] EMBOSS, The European Molecular Biology Open Software Suite. http://emboss.sourceforge.net/what/.

[B47] Vega Laso MR, Zhu D, Sagliocco F, Brown AJ, Tuite MF, McCarthy JE (1993). Inhibition of translational initiation in the yeast *Saccharomyces cerevisiae *as a function of the stability and position of hairpin structures in the mRNA leader. J Biol Chem.

[B48] Kozak M (1994). Determinants of translational fidelity and efficiency in vertebrate mRNAs. Biochimie.

[B49] Hughes DJ, Ginolhac SM, Coupier I, Corbex M, Bressac-de-Paillerets B, Chompret A, Bignon YJ, Uhrhammer N, Lasset C, Giraud S (2005). Common BRCA2 variants and modification of breast and ovarian cancer risk in BRCA1 mutation carriers. Cancer Epidemiol Biomarkers Prev.

[B50] Hu N, Li WJ, Su H, Wang C, Goldstein AM, Albert PS, Emmert-Buck MR, Kong LH, Roth MJ, Dawsey SM (2003). Common genetic variants of TP53 and BRCA2 in esophageal cancer patients and healthy individuals from low and high risk areas of northern China. Cancer Detect Prev.

[B51] Khor CC, Chapman SJ, Vannberg FO, Dunne A, Murphy C, Ling EY, Frodsham AJ, Walley AJ, Kyrieleis O, Khan A (2007). A Mal functional variant is associated with protection against invasive pneumococcal disease, bacteremia, malaria and tuberculosis. Nat Genet.

[B52] Healey CS, Dunning AM, Teare MD, Chase D, Parker L, Burn J, Chang-Claude J, Mannermaa A, Kataja V, Huntsman DG (2000). A common variant in BRCA2 is associated with both breast cancer risk and prenatal viability. Nat Genet.

[B53] Moynahan ME, Pierce AJ, Jasin M (2001). BRCA2 is required for homology-directed repair of chromosomal breaks. Mol Cell.

[B54] Arnold K, Kim MK, Frerk K, Edler L, Savelyeva L, Schmezer P, Wiedemeyer R (2006). Lower level of BRCA2 protein in heterozygous mutation carriers is correlated with an increase in DNA double strand breaks and an impaired DSB repair. Cancer Lett.

[B55] Lee SA, Baker MD (2007). Analysis of DNA repair and recombination responses in mouse cells depleted for Brca2 by SiRNA. DNA Repair (Amst).

