# Supplementary material for: Implication of BRCA2 -26G>A 5' untranslated region polymorphism in susceptibility to sporadic breast cancer and its modulation by p53 codon 72 Arg>Pro polymorphism
Source: Breast Cancer Res. 2007 Oct 18;9(5):R71. doi: 10.1186/bcr1780 (PMC2242669; doi:10.1186/bcr1780)
Supplement: Additional file 1 — Details of the somatic mutations found in the exons 5-8 (DNA binding domain) of the p53 gene in sporadic breast cancer patients [file bcr1780-S1.doc]

**Supplementary Table 1**

**Details of the somatic mutations found in the exons 5-8 (DNA binding domain) of the p53 gene in sporadic breast cancer patients**

| **SL. NO.** | **EXON /INTRON** | **NT. NO.^1^** | **CODON NO.** | **MUTATION (HUGO)^2^** | **BASE CHANGE^3^** | **AMINO ACID CHANGE** | **EFFECT** |
| --- | --- | --- | --- | --- | --- | --- | --- |
|
|
|  |  |  |  |  |  |  |  |
| 1 | 5 | 13113 | 145 | 1397T>A | CTG>CAG | Leu>Gln | MS |
| 2 | 5 | 13130 | 151 | 1414C>T | CCC>TCC | Pro>Ser | MS |
| 3 | 5 | 13167 | 163 | 1451A>G | TAC>TGC | Tyr>Cys | MS |
| 4 | 5 | 13172-13175 | 165-166 | 1456-1459 delCAGT | CAG TCA | - | FS |
| 5 | 5 | 13178 | 167 | 1462C>T | CAG>TAG | Gln>STOP | NS |
| 6 | 5 | 13203 | 175 | 1487G>A | CGC>CAC | Arg>His | MS |
| 7 | 5 | 13203 | 175 | 1487G>A | CGC>CAC | Arg>His | MS |
| 8 | 6 | 13338 | 193 | 1622A>G | CAT>CGT | His>Arg | MS |
| 9 | 6 | 13346 | 196 | 1630C>T | CGA>TGA | Arg>STOP | NS |
| 10 | 6 | 13397 | 213 | 1681C>T | CGA>TGA | Arg>STOP | NS |
| 11 | 6 | 13403 | 215 | 1687A>C | AGT>CGT | Ser>Arg | MS |
| 12 | 6 | 13406 | 216 | 1690G>A | GTG>ATG | Val>Meth | MS |
| 13 | 6 | 13419 | 220 | 1703A>G | TAT>TGT | Tyr>Cys | MS |
| 14 | 7 | 14034 | 236 | 2318A>G | TAC>TGC | Tyr>Cys | MS |
| 15 | 7 | 14034 | 236 | 2318A>G | TAC>TGC | Tyr>Cys | MS |
| 16 | 7 | 14045 | 240 | 2329A>T | AGT>TGT | Ser>Cys | MS |
| 17 | 7 | 14052 | 242 | 2336G>A | TGC>TAC | Cys>Tyr | MS |
| 18 | 7 | 14051 &14052 | 242 | 2335T>C, 2336G>T | TGC>CTC | Cys>Leu | MS |
| 19 | 7 | 14061 | 245 | 2345G>A | GGC>GAC | Gly>Asp | MS |
| 20 | 8 | 14458 | 263 | 2742T>G | AAT>AAG | Asn>Lys | MS |
| 21 | 8 | 14465 | 266 | 2747G>A | GGA>AGA | Gly>Arg | MS |
| 22 | 8 | 14487 | 273 | 2771G>A | CGT>CAT | Arg>His | MS |
| 23 | 8 | 14487 | 273 | 2771G>A | CGT>CAT | Arg>His | MS |
| 24 | 8 | 14510 | 281 | 2794G>C | GAC>CAC | Asp>His | MS |
| 25 | 8 | 14517 | 283 | 2802G>C | CGC>CCC | Arg>Pro | MS |
|  |  |  |  |  |  |  |  |

^1^Transcription initiation site as +1 nucleotide. ^2^Mutations defined according to Human Genome Organization (HUGO) nomenclature using translation initiation site as +1 nucleotide. ^3^Mutated nucleotide underlined. SL.NO.,serial number; MS, missense; FS, frameshift; NS, nonsense.
