# Supplementary material for: Implication of BRCA2 -26G>A 5' untranslated region polymorphism in susceptibility to sporadic breast cancer and its modulation by p53 codon 72 Arg>Pro polymorphism
Source: Breast Cancer Res. 2007 Oct 18;9(5):R71. doi: 10.1186/bcr1780 (PMC2242669; doi:10.1186/bcr1780)
Supplement: Additional file 2 — Summary of p53 codon 72 association studies in breast cancer across the globe [file bcr1780-S2.doc]

Supplementary Table 2

**Summary of p53 codon 72 association studies in breast cancer across the globe**

| **Ethnicity/Country** | **PRO/PRO (%)** | **PRO/ARG (%)** | **ARG/ARG (%)** | **REFERENCE NO.** |  | **Ethnicity/Country** | **PRO/PRO (%)** | **PRO/ARG (%)** | **ARG/ARG (%)** | **REFERENCE** |
| --- | --- | --- | --- | --- | --- | --- | --- | --- | --- | --- |
| **SWEDISH** |  |  |  |  |  | **NORTH INDIA** |  |  |  |  |
| PATIENTS (212) | 11.3 | 43.9 | 44.8 |  |  | PATIENTS (243) | 19.8 | 44.9 | 35.4 |  |
| CONTROLS (689) | 8.9 | 36.7 | 54.4 | [26] |  | CONTROLS (333) | 29.1 | 48.0 | 22.8 | Present study |
| **CAUCASIANS** |  |  |  |  |  | **AFRICAN AMERICAN** | |  |  |  |
| PATIENTS (65) | 9.2 | 41.5 | 49.2 |  |  | PATIENTS (16) | 37.5 | 56.3 | 6.3 |  |
| CONTROLS (117) | 2.6 | 35.9 | 61.5 | [27] |  | CONTROLS (30) | 40.0 | 46.7 | 13.3 | [27] |
| **CHUBU, JAPAN** |  |  |  |  |  | **INDIA** |  |  |  |  |
| PATIENTS (200) | 18.0 | 50.0 | 32.0 |  |  | PATIENTS (77) | 7.8 | 66.2 | 26.0 |  |
| CONTROLS (282) | 10.7 | 48.9 | 40.4 | [28] |  | CONTROLS (41) | 19.5 | 58.5 | 22.0 | [34] |
| **HISPANIC** |  |  |  |  |  | **TUNISIA** |  |  |  |  |
| PATIENTS (18) | 16.7 | 44.4 | 38.9 |  |  | PATIENTS (30) | 10.0 | 30.0 | 60.0 |  |
| CONTROLS (38) | 26.3 | 42.1 | 31.6 | [27] |  | CONTROLS (49) | 8.2 | 53.1 | 38.8 | [35] |
| **GREECE** |  |  |  |  |  | **RUSSIA** |  |  |  |  |
| PATIENTS (56) | 21.0 | 18.0 | 61.0 |  |  | PATIENTS (529) | 7.9 | 38.4 | 53.7 |  |
| CONTROLS (61) | 10.0 | 67.0 | 26.0 | [29] |  | CONTROLS (420) | 6.4 | 37.9 | 49.3 | [36] |
| **TURKEY** |  |  |  |  |  | **OSAKA, JAPAN** |  |  |  |  |
| PATIENTS (115) | 10.4 | 33.9 | 55.6 |  |  | PATIENTS (191) | 15.2 | 36.1 | 48.7 |  |
| CONTROLS (76) | 15.8 | 56.5 | 15.8 | [30] |  | CONTROLS (218) | 14.2 | 34.9 | 50.9 | [37] |
| **NORTHERN GREECE** | |  |  |  |  | **JORDAN** |  |  |  |  |
| PATIENTS (42) | 7.0 | 31.0 | 62.0 |  |  | PATIENTS (43) | 18.6 | 44.2 | 37.2 |  |
| CONTROLS (51) | 17.0 | 63.0 | 20.0 | [31] |  | CONTROLS (47) | 10.6 | 55.3 | 34.0 | [38] |
| **ASHKINAZI (JEWISH)** | |  |  |  |  | **FINNISH** |  |  |  |  |
| PATIENTS (108) | 1.9 | 29.6 | 68.5 |  |  | PATIENTS (1551) | 7.0 | 39.8 | 53.2 |  |
| CONTROLS (60) | 10.0 | 40.0 | 50.0 | [32] |  | CONTROLS (733) | 7.1 | 37.9 | 55.0 | [39] |
| **NON-ASHKINAZI (JEWISH)** | |  |  |  |  | **SHIRAZ, IRAN** |  |  |  |  |
| PATIENTS (24) | 4.2 | 33.3 | 62.5 |  |  | PATIENTS (221) | 13.1 | 49.3 | 37.6 |  |
| CONTROLS (107) | 12.2 | 65.4 | 22.4 | [32] |  | CONTROLS (205) | 19.5 | 43.9 | 36.6 | [40] |
| **PORTO ALEGRE, SOUTHERN BRAZIL** | | |  |  |  | **BOSTON, USA (CAUCASIAN)** | |  |  |  |
| PATIENTS (118) | 5.1 | 40.7 | 54.2 |  |  | PATIENTS (1477) | 7.0 | 38.5 | 54.4 |  |
| CONTROLS (202) | 10.3 | 54.9 | 34.6 | [33] |  | CONTROLS (2224) | 5.9 | 37.7 | 56.4 | [41] |
|  |  |  |  |  |  |  |  |  |  |  |

Pro/Pro association Arg/Arg association No association
